# Breathable, Adhesive, and Biomimetic Skin‐Like Super Tattoo

**DOI:** 10.1002/advs.202406706

**Published:** 2024-08-29

**Authors:** Chuqi Li, Zhiyuan Tan, Xiaohu Shi, Dekui Song, Yan Zhao, Yan Zhang, Zihan Zhao, Weifeng Zhang, Jiongyang Qi, Yifang Wang, Xin Wang, Zhenquan Tan, Nan Liu

**Affiliations:** ^1^ Beijing Key Laboratory of Energy Conversion and Storage Materials, College of Chemistry Beijing Normal University Beijing 100875 P. R. China; ^2^ State Key Laboratory of Fine Chemicals, Panjin Branch of School of Chemical Engineering Dalian University of Technology Panjin Liaoning 124221 P. R. China

**Keywords:** biomimetic, skin‐like tattoo, ultra‐thin, universal platform

## Abstract

Electronic tattoo, capable of imperceivably acquiring bio‐electrical signals from the body, is broadly applied in healthcare and human‐machine interface. Tattoo substrate, the foundation of electronic tattoo, is expected to be mechanically mimetic to skin, adhesive, and breathable, and yet remains highly challenging to achieve. Herein, the study mimics human skin and design a breathable, adhesive, and mechanically skin‐like super tattoo substrate based on an ultra‐thin film (≈2 µm). Similar to skin, super tattoo demonstrates strain‐adaptive stiffening properties with high tear energy (5.4 kJ·m^−2^) and toughness (1.3 MJ·m^−3^). Superior to skin, it exhibits high adhesion, ionic conductivity, and permeability. A variety of conductive electrodes can be processed on it, showing the universality toward an ideal platform for electronic tattoo with stable and low contact impedance. Super tattoo‐based electrodes can imperceivably and accurately monitor weak electromyography (EMG) of swallowing on the junction, providing effective guidance for rehabilitation training of dysphagia.

## Graphical abstract

Super tattoo is constructed by mimicking the structure of skin whose fibrous scaffold is embedded in the matrix, demonstrating strain‐stiffening property as skin with high tear energy and toughness. Superior to skin, the ultrathin tattoo (≈2 µm) exhibits high adhesion, ionic conductivity, and breathability which can replace EC tattoo as a new general platform for bioelectrodes.

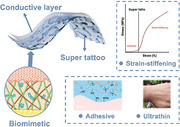

## 1 Introduction

Electronic skin refers to wearable electronics mounted on skin that sense body signals for a variety of applications, such as mobile healthcare, sport training, human‐machine interface and etc.^[^ ^1^ ^]^ It is anticipated to propel technological advancements forward toward greater efficiency, comfort and healthiness. However, given that human skin secretes sweat to regulate temperature and maintain water‐salt balance, the excessive thickness of electronic skin can impede the evaporation of surface moisture, causing discomfort and even inflammation.^[^ ^2^ ^]^ Consequently, there is a growing demand for self‐adaptive electronic skin that not only spontaneously adhere to skin but also maintains breathability and moisture permeability.^[^ ^3^ ^]^

Tattoo substrates, such as ethyl cellulose (EC) tattoos, have been predominately employed for this purpose. Their ultra‐thin thickness enables conformal contact with skin, but the dense structure of the polymer substrate still inevitably hinders the evaporation of water.^[^ ^2^, ^4^ ^]^ Moreover, when the skin undergoes deformation, such as swallowing and chewing in the throat, excessive stretching can lead to device damage. Therefore, it is imperative to develop a novel tattoo substrate that is ultrathin to be conformal on skin as well as mechanically matches with skin,^[^ ^5^ ^]^ regardless of whether it is at rest or undergoing large deformations. Such a substrate would significantly contribute to the advancement of electronic skin technology.

To tackle this problem, we propose to study skin tissue and develop a biomimetic super tattoo substrate, which can be similar to skin in nature and function for imperceivable and high‐performance sensing bioelectrical signals. Skin, composed of three layers, including epidermis, dermis and hypodermis,^[^ ^6^ ^]^ is the largest organ in our body, which can produce oil or sweat, protect against bacteria or germs, and sense external forces, temperature and etc. Mechanical properties and sensing capability are mainly determined by the thickest dermal layer.^[^ ^7^ ^]^ The dermis with mutual promotion consists of collagen fibers and the gel matrix.^[^ ^8^ ^]^ The intertwined 3D collagen fiber network is primarily responsible for conferring tensile strength and structural support to the skin tissue.^[^ ^9^ ^]^ The matrix, filled with the network, endows the skin with exceptional viscosity. Furthermore, the polysaccharides present in the matrix, such as hyaluronic acid, are rich in positive and negative charges, thereby imparting excellent ion mobilizing and water moisturizing properties.^[^ ^10^ ^]^ Serving as a “brick tile”, the matrix prevents protein fibers from dehydration and brittleness, and also facilitates the transmissions of water and ion electrical signals, enabling the skin to function as a biosensor.^[^ ^11^ ^]^ Their mechanical property is very unique, presenting a J‐shaped stress‐strain curve with strain stiffening characteristic, making the skin soft on touch and stiff upon deformation.^[^ ^9^ ^]^ The structure of high modulus fibers embedded in low modulus matrix also helps to improve the skin's tear resistance.^[^ ^12^ ^]^ Inspired by human skin, several polymers have been designed to replicate the mechanical and electrical properties of skin tissue. Sergei group mimicked chameleon and synthesized liner‐bottlebrush‐linear triblock copolymers with synergistic capacity for strain‐adaptive stiffening and coloration.^[^ ^9^ ^]^ Wu group resembled the repairable and fatigue‐resistant human skin and designed a hybrid ionic skin composed of self‐healing polyurethane nanomesh and supramolecular ionic matrix.^[^ ^13^ ^]^ These works demonstrated excellent strategies for synthesizing skin‐like polymer, and yet the ability to integrate skin properties and ultrathin breathable feature into electronic tattoo remains elusive.

Herein, by replicating the structure of human skin,^[^ ^14^ ^]^ we synthesized an ultrathin (≈2 µm) skin‐like super tattoo substrate. And leveraging its breathability, adhesiveness, mechanical and electrical properties that mimicked those of skin, we further developed it into a general platform for electronic tattoo,^[^ ^15^ ^]^ enabling the imperceivable and accurate monitoring of very weak electrophysiological signals both when the skin is at rest or undergoing large deformations. Swallowing, as one of our most common behaviors in our daily life, accompanies with large skin deformation between head and neck muscles. Globally, half a billion people suffer from dysphagia caused by stroke, cerebral palsy and Parkinson's disease. To assist those patients with swallowing disorders, we have applied our electronic tattoo on the junction of the mandibular triangle and neck, and monitored electromyography (EMG) during swallowing for rehabilitation training and treatment that improve their swallowing function. Our newly developed skin‐like super tattoo is expected to boost the development of electronic tattoo, establishing a robust and versatile foundation for self‐adaptive electronic skin.

## 2 Results and Discussion

### 2.1 Design of Breathable, Adhesive, and Biomimetic Skin‐Like Super Tattoo

**Figure 1:**
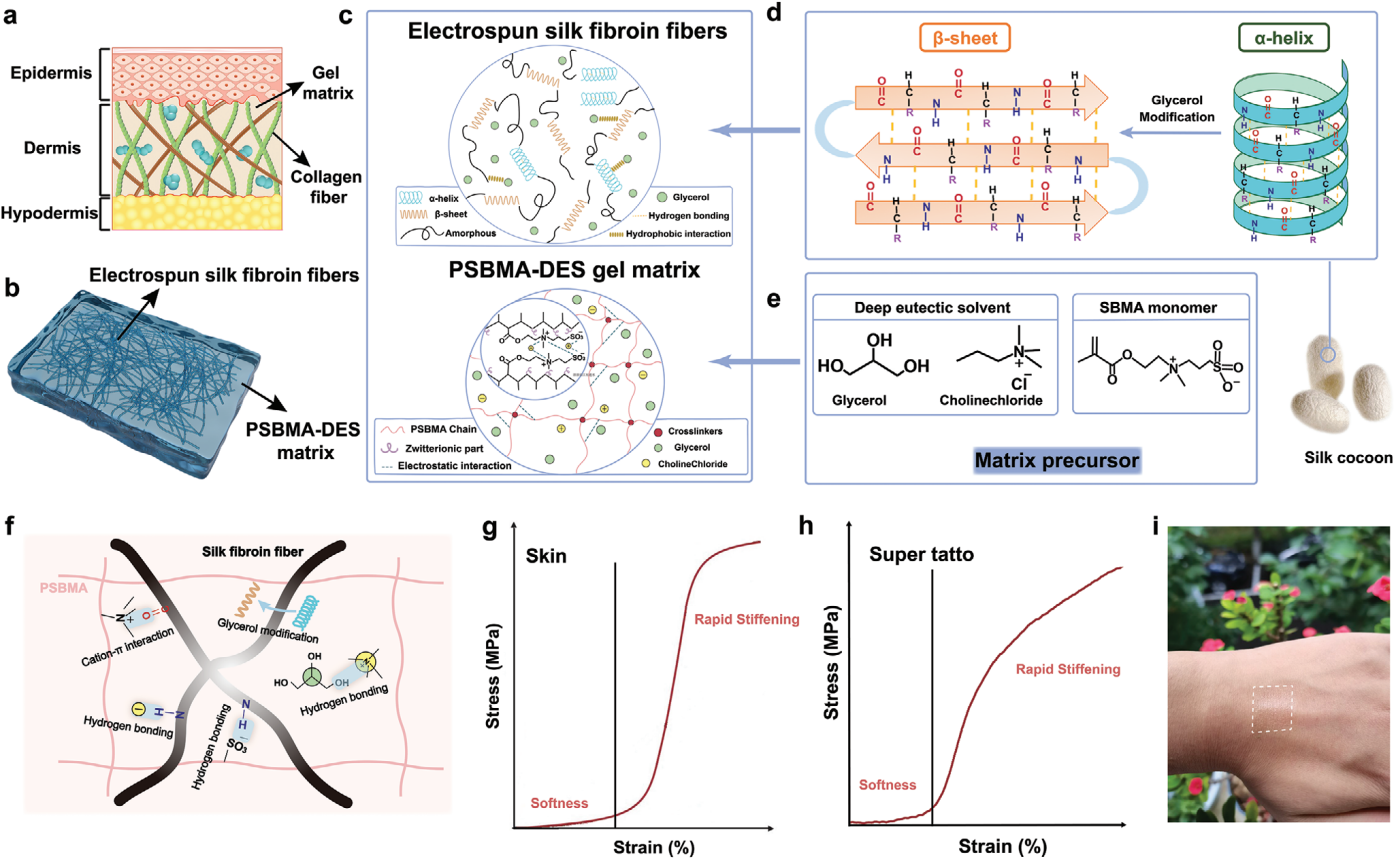
Design of breathable, adhesive, and biomimetic skin‐like super tattoo. a) Microstructure of human skin. b) Designed microstructure of super tattoo, consisting of electrospun silk fibroin fibers and zwitterionic gel (PSBMA) polymerized in deep eutectic solvent (DES) as matrix. c) Chemical interactions present in electrospun silk fibroin fibers and PSBMA‐DES gel matrix. d) Schematic illustration of the secondary structure evolution. e) The components used for preparation of matrix precursor. f) Schematic diagram of the synergistic effect between the electrospun silk fibroin fibers and PSBMA‐DES gel matrix. g) Stress–strain curve of skin. h) Super tattoo with representative J‐shaped characteristics. i) Photos of super‐tattoo adhering on hand.

To establish a universal platform for electronic tattoo, we emulated the intricate structure of human skin tissue (**Figure** **1**a), fabricated a skin‐like super tattoo (Figure 1b,c), and imparted it breathability and adhesiveness for imperceivable and conformal contact even when skin is under deformation. Leveraging the massive production, excellent mechanical properties, biodegradability and biocompatibility of silk, electrospun silk fibroin fibers were used as the replication of collagen fibers. Glycerol was incorporated as a plasticizer to interact with hydrophobic part of silk fibroin and effectively increased the β‐sheet content^[^ ^16^ ^]^ (Figure 1d). It also served as a surrogate for water during the hydration of silk fibroin chains to improve the stability of the helical structures of silk fibroin. Consequently, the resulted electrospun silk fibroin fibers exhibit enhanced fracture strength, tensile elasticity and air durability, rendering them resilient to subsequent processing steps and practical use. Taking advantages of the self‐charge and hydrophilic properties of zwitterionic polymer and deep eutectic solvent (DES), SBMA ([2‐(methacryloyloxy) ethyl] dimethyl‐(3‐sulfopropyl) ammonium hydroxide) polymerized in DES (1:2 choline chloride: glycerol) was applied as the matrix for the super tattoo (Figure 1e). Electrospun silk fibroin fibers were immersed in the matrix precursor and in situ photopolymerized using a dip‐coating‐photopolymerization process (Figure S1d, Supporting Information). Analogous to collagen fiber and matrix in skin, electrospun silk fibroin fibers and PSBMA‐DES gel matrix also display synergistic effects (Figure 1f). Silk fibroin fibers, with their enhanced mechanical property, serve as a robust framework, supporting the soft PSBMA‐DES gel matrix as an ultra‐thin and breathable film. On the other hand, the impregnated PSBMA‐DES gel, enriched in abundant sulfonic acid and ammonium cationic groups, and positive and negative charges, which may further plasticize and stabilize silk fibroin fibers by dynamic hydrogen bonding and electrostatic interaction. Ultimately, super tattoo mimicked the mechanical and electrical property of skin, exhibiting a J‐shaped stress‐strain curve and ionic conductivity (Figure 1g,h). In contrast to EC tattoo, super tattoo adhered seamlessly on skin regardless of rest and deformed states, due to the ultrathin nature and adhesive property (Figure 1i). It can also be worn on skin for many days without causing sweat accumulation, skin redness and itching, reflecting its breathability and biocompatibility. Therefore, super tattoo stands as an excellent platform for self‐adaptive electronic tattoo, offering breathability, adhesiveness and biomimetic characteristics.

### 2.2 Fabrication and Characterization of Super Tattoo

**Figure 2:**
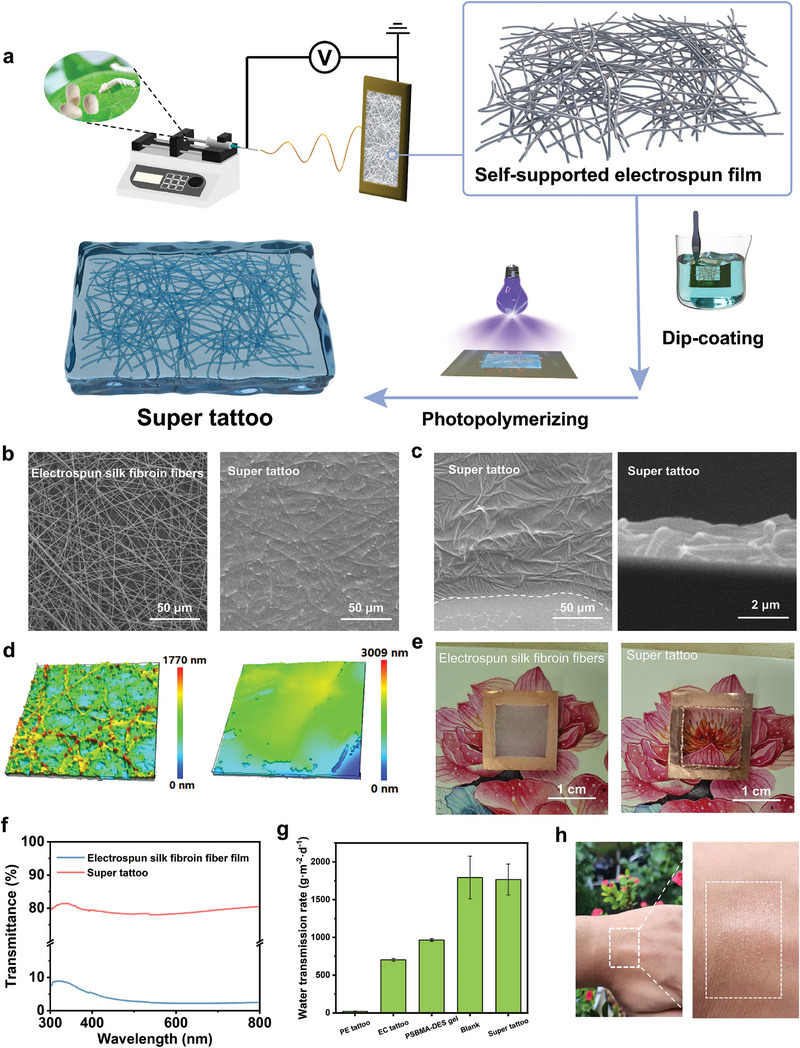
Fabrication and characterization of super tattoo. a) Fabrication process of super tattoo involving electrospinning silk fibroin fibers and dip‐coating‐photopolymerizing PSBMA‐DES gel. b) SEM images of electrospun silk fibroin fibers and as‐covered PSBMA‐DES gel forming into super tattoo. c) SEM images on the edge of super tattoo (left) and its cross‐section view (right). d) White light interferometer optical images of electrospun silk fibroin fiber film (left) and super tattoo (right). e) Photos of electrospun silk fibroin fiber film (left) and super tattoo (right) on Cu frames. f) Ultraviolet‐visible spectra (spectral range from 300 nm to 800 nm) of electrospun silk fibroin fiber film (blue) and super tattoo (red). g) Comparison of the water vapor transmission rate of different thin films. h) Photos of super tattoo attaching to skin, showing its transparency.

Fabrication of super tattoo involved two steps: 1) electrospinning silk fibroin fibers and 2) dip‐coating precursor solution and photopolymerization into PSBMA‐DES gel (**Figure** **2**a). Morphology characterization was first performed by scanning electron microscope (SEM) with samples on Si substrates. Solvent composition, silk fibroin concentration and electrospinning condition all affect the formation of silk fibroin fibers.^[^ ^17^ ^]^ Viscoelastic property of the electrospinning solution greatly influences the morphology of as‐achieved fibers, because the molecular chains need to entangle at an appropriate degree to resist external tensile force or aggregation of the spinning jet (Figure S2, Supporting Information). After wrapping PSBMA‐DES gel matrix precursor onto electrospun silk fibroin fibers, in situ polymerization was initiated by 365 nm UV light, resulting in PSBMA‐DES gel as a thin matrix (Figure 2b). The tilted view of super tattoo indicated the ultra‐thin characteristic of gel layer, whose edge maintained close contact on the Si substrate. From cross‐section view, the thickness of super tattoo was estimated to be in micrometer (µm) scale (Figure 2c). As super tattoo possesses large amount of hydrogen bonding, its surface is very adhesive, rendering it challenging to be characterized effectively using tapping mode or contact mode atomic force microscopy. Instead, white light diffractometer was employed to observe their surface morphology, revealing that the thickness of super tattoo is ≈2 µm. Moreover, a notable reduction in roughness was observed after in situ photopolymerization (Figure 2d). Less‐rough surface can reduce the refraction and scattering of intermittent light, which leads to an increase in the transmittance. For easy handling and following processing, electrospun silk fibroin film and super tattoo were directly fabricated on Cu frames. It was observed that the former silk fibroin film was opaque, while the latter super tattoo was transparent (Figure 2e). The transparency of pure electrospun silk fibroin fiber film and super tattoo at 550 nm were ≈2% and ≈78% respectively (Figure 2f). This observation aligned with the roughness and thickness measurements through white light diffractometer analysis.

The secretion of sweat often disrupts the stability of contact between the electronic tattoo and the skin, leading to electrical signal acquisition interferences. Excessive accumulation of sweat can also irritate the skin, causing rashes or inflammation.^[^ ^13^, ^18^ ^]^ Our ultrathin and transparent super tattoo is highly likely to avoid above impact of sweat due to its breathability. Water vapor transmission experiments, which were conducted using the sealed water bottle method (Figure S3a, Supporting Information), revealed that the water vaper transmission rate (WVTR) of super tattoo (1767 g·m^−2^·d^−1^) was close to that of an open bottle (1793 g·m^−2^·d^−1^) and significantly higher than that of standard PE tattoo (20 g·m^−2^·d^−1^) and commercial EC tattoo (704 g·m^−2^·d^−1^) (Figure 2g). The exceptional water permeability of super tattoo was attributed to the grid structure of electrospun silk fibroin fiber film. Furthermore, the zwitterion group, glycerol and choline chloride present on the molecular chain of PSBMA‐DES gel possessed strong moisture absorption capability,^[^ ^19^ ^]^ enabling it to effectively capture water molecules and facilitate their transfers to the exterior, thus maintaining a balanced water level at the skin interface and preventing sweat from accumulating on skin. Overall, our ultrathin, transparent and breathable super tattoo (≈2 µm in thick) will not only enhance the aesthetics and comfort of wearability, but also potentially serve as an exceptional platform for electronic skin applications (Figure 2h).

### 2.3 Skin‐Like Tensile Properties of Super Tattoo

**Figure 3:**
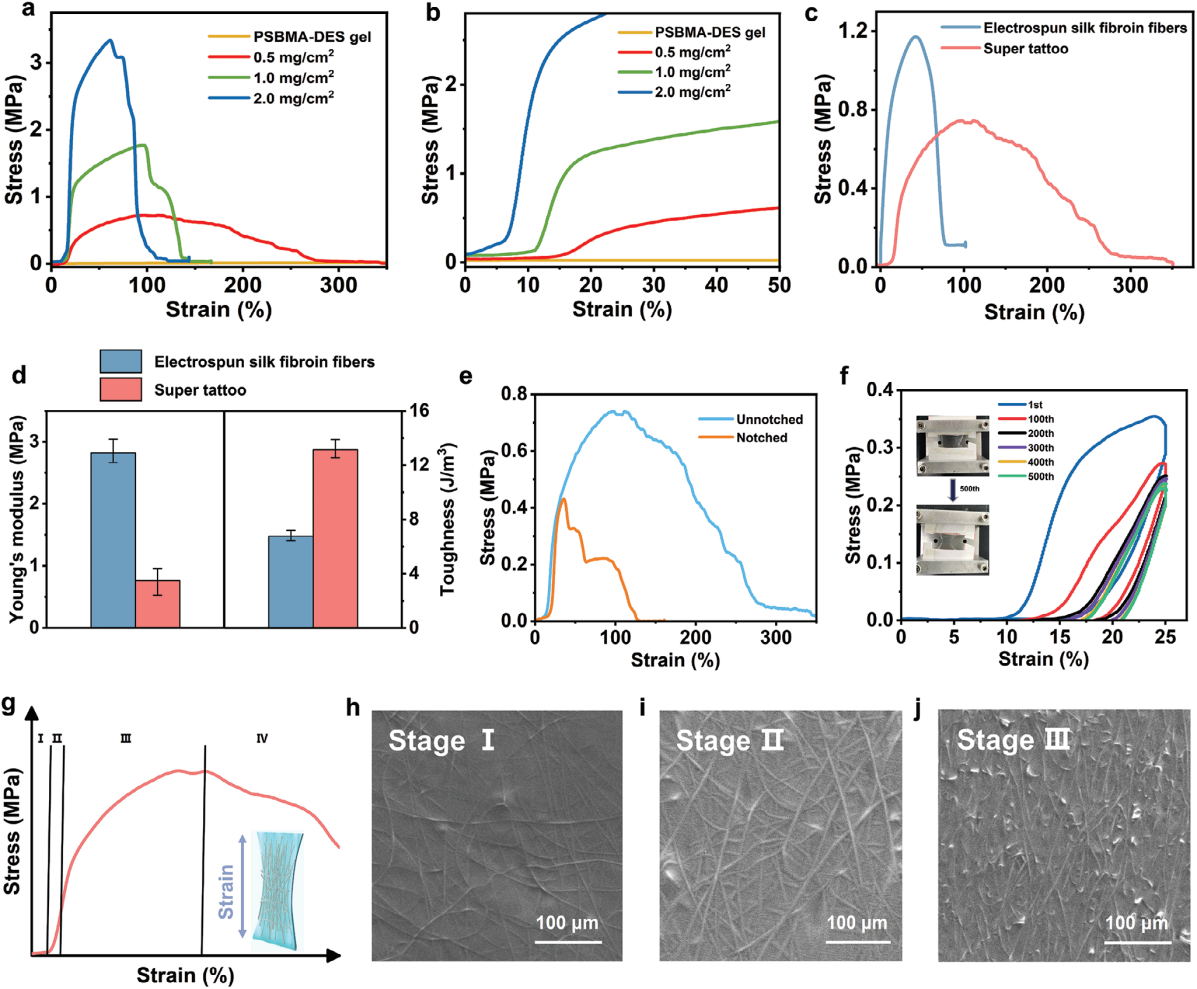
Skin‐like tensile properties of super tattoo. a,b) Stress–strain curves of super tattoo films with different densities of electrospun silk fibroin fibers (a), and their enlarged stress–strain curves (b) showing strain stiffening characteristics. c,d) Stress–strain curves of plain electrospun silk fibroin fiber film and super tattoo film after covering PSBMA‐DES gel (c), and corresponding comparison of Young's modulus and toughness (d). e) Stress–strain curves of unnotched and notched super tattoo. f) Cycled tensile‐release curves of notched super tattoo for 500 cycles (inset: photos of the notched super tattoo before and after 500th cycled stretching up to 25% strain). g) Representative stress‐strain curve of super tattoo corresponding to status of softness, transition, stiffening, and declining labeled as stage I, II, III, IV respectively. h–j) SEM images of super tattoo during stretching corresponding to stage h) I, i) II, j) III.

The skin has a unique fiber‐matrix structure, which exhibits nonlinear strain stiffening behavior due to the presence of fibers in the matrix, presenting a unique J‐shaped stress‐strain curve.^[^ ^9^, ^12^, ^20^ ^]^ It has a lower Young's modulus at low strain to ensure good skin deformation. As the strain increases, the Young's modulus of the skin rapidly increases, playing a protective and defensive role on human tissues. Super tattoo has a similar strain hardening behavior. Mechanical tensile tests were performed on pure super tattoo film, where were suspended on Cu frames (**Figure** **3**a; Figure S4, Supporting Information). In the absence of electrospun silk fibroin fibers, PSBMA‐DES gel is very soft (Figure S5, Supporting Information) with an extremely low tensile strength and Young's modulus. Conversely, the integration of electrospun silk fibroin fibers transformed the super tattoo film into robust materials, exhibiting tensile curves reminiscent of skin's J‐shaped curve (Figure 3b). Furthermore, an increase in the density of these fibers leaded to a steeper slope in strain stiffening with a reduced onset strain. The skin‐like tensile behavior of super tattoo was also caused by its fiber‐matrix structure. Plain electrospun silk fibroin fiber film demonstrated a higher Young's modulus (>2.5 MPa) but lacks ductility (<50%). After skinning PSBMA‐DES gel, super tattoo exhibited a lower Young's modulus and larger toughness (1.3 ± 0.2 MJ·m^−3^) (Figure 3c,d). The toughness of super tattoo can be attributed to the plasticizing effect of PSBMA‐DES gel on electrospun silk fibroin fibers. The gel, consisted of polyzwitterion, glycerol and choline chloride, possessed excellent water absorption capabilities. This absorption disrupted the hydrogen bonds within the amorphous structure of silk fibroin, weakening the intermolecular interaction and enhancing its liquidity.^[^ ^21^ ^]^ Thus, plasticization of silk fibroin fiber boosted the toughness of super tattoo. Interestingly, the stress‐strain curve of super tattoo at the point of fracture showed a gradual, plastic‐like decline, rather than a “cliff” drop‐off. And with the decreased density of silk fibroin fibers, the plastic decline became even more gradual. By combining the J‐shaped curve with the trailing characteristics, it is hypothesized that fibers within super tattoo primarily determined its tensile strength, while the gel matrix softened the entire material, conferring high ductility and low modulus.

Super tattoo, with a crack spanning one‐fifth of its width, underwent further tensile testing to assess its fracture resistance (Figure 3e). During stretching, the notch was passivated, enabling the notched super tattoo to retain a significant amount of stress and strain. Specifically, it remained 58% stress and 46% strain compared to unnotched samples. The fracture energy of super tattoo calculated using the unilateral notch tension method was as high as 5.4 kJ·m^−2^, which was higher than the fracture energy of skin^[^ ^12a^ ^]^ at 2 kJ·m^−2^. Additionally, the fatigue resistance of super tattoo was measured using cyclic tensile notch samples, and the crack did not extend significantly after 500 cycles at 25% strain (Figure 3f). A high fatigue threshold of 236 J·m^−2^ was calculated, further testament to the material's durability and resilience.

Morphology evolution upon strain was systematically analyzed to reveal the mechanism of skin‐like tensile properties of super tattoo (Figure 3g). Initially, electrospun silk fibroin fibers in super tattoo were disordered (strain: 0%). As the film stretched, the fibers gradually drew closer to each other. During this process, the fibers primarily underwent spatial deformation, which did not contribute much to the tensile strength. This corresponded to the first stage of low modulus in the J‐shaped curve, specifically marked as Stage I. As strain was continuously applied to the film, the fibers drew closer together, gradually leading to increase in the film's modulus. The fibers themselves underwent slight deformation and gradually commenced bearing stress. The stress‐strain curve began to show inflection points, marking the transition to the second stage (Stage II). The film continued to be stretched until the fiber orientation reached a high degree of consistency. At this stage, the fibers bore the main stress, and the film stiffens, corresponding to the third stage of high modulus (Stage III). To observe fracture process and estimate fracture resistance, a small notch perpendicular to the strain direction was introduced. Before the fiber breaks at the notch edge, spatial deformation will occur, increasing the orientation and squeezing the gel to fill voids. This process stiffened the fiber edge, preventing crack propagation and making the crack passivate. When a fiber ultimately broke, the energy accumulated within the film was dissipated. This stress dissipation caused the notched super tattoo to exhibit a stepped fracture curve rather than a cliff drop‐off, leading super tattoo to the high tear toughness. Thus, super tattoo can still maintain a certain degree of strain after yielding, avoiding fracture at excessive strain.

### 2.4 Adhesion and Ion Conductivity of Super Tattoo

**Figure 4:**
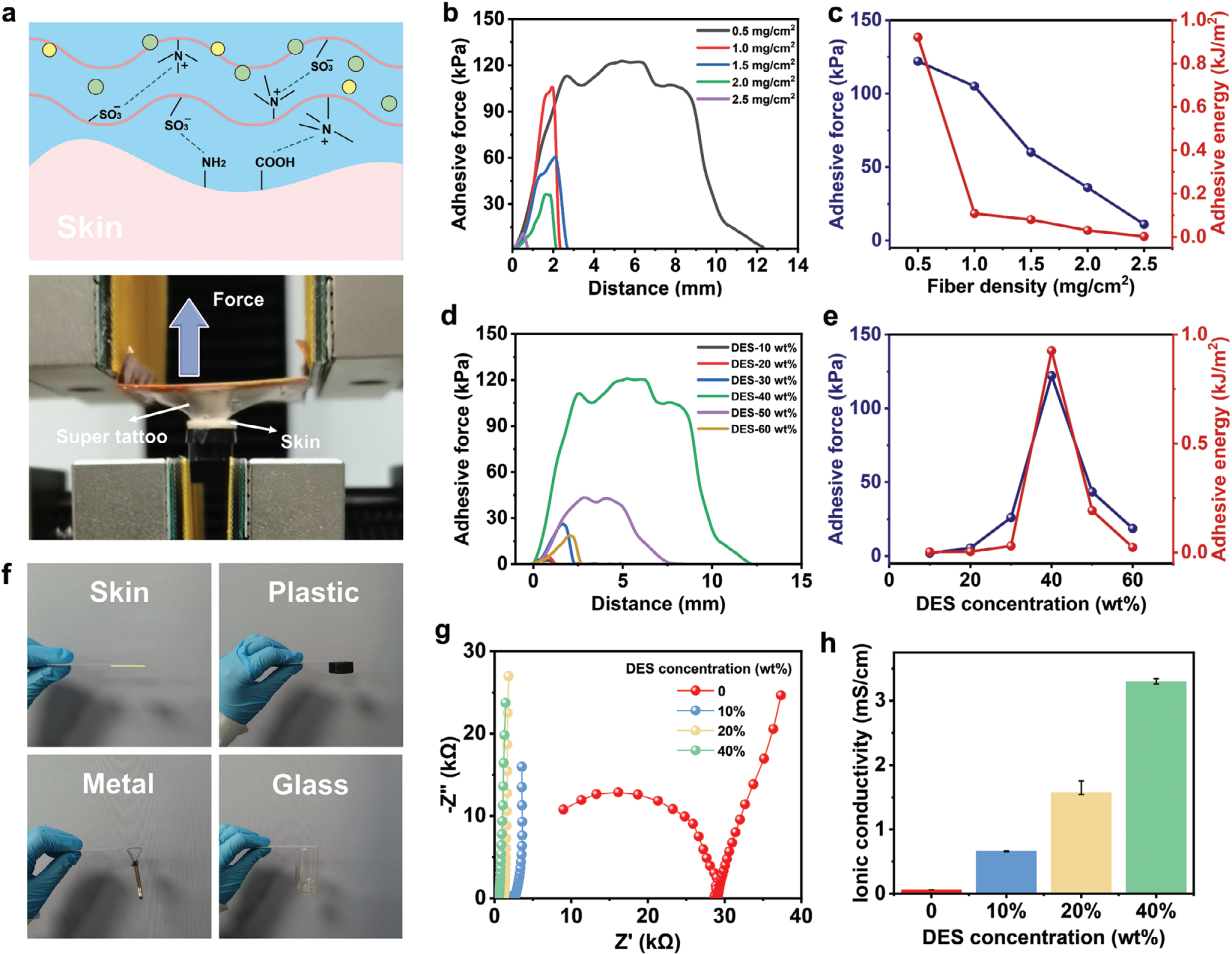
Adhesion and ion conductivity of super tattoo. a) Schematic diagram of the adhesive mechanism of super tattoo (above), and a photo of an adhesive, free‐standing super tattoo detached from skin during a tack separation experiment (below). b) Force stroke curves of super tattoo adhered to skin with different densities of electrospun silk fibroin fibers. c) Comparison of maximum adhesive force and adhesive energy of super tattoo with different densities of electrospun silk fibroin fibers. d) Force stroke curves of super tattoo adhered to skin with different DES concentrations. e) Comparison of maximum adhesive force and adhesive energy of super tattoo with different DES concentrations. f) Photos of super tattoo adhered to skin, plastic, metal, and glass. g) Nyquist plots of super tattoo with different DES concentrations. h) Ion conductivity of super tattoo with different DES concentrations.

In addition to ultrathin thickness, adequate adhesion on skin is crucial for electronic tattoo to eliminate interface gaps^[^ ^22^ ^]^ and accurately sense bio‐signals.^[^ ^23^ ^]^ Compared to EC tattoo, the adhesiveness of super tattoo is another advantage as a tattoo substrate. SBMA consists of sulfonyl and quaternary ammonium salt groups and DES is composed of hydrogen bond acceptors and hydrogen bond donors.^[^ ^24^ ^]^ The abundant dynamic bonding, including hydrogen bonding, ion interaction and van der Waals force co‐determined the adhesion between super tattoo and skin^[^ ^25^ ^]^ (**Figure** **4**a). The adhesive force on skin was evaluated by tack separation experiment and the effects of electrospun silk fibroin fibers and PSBMA‐DES gel were explored. As the fiber density increased, the adhesive force and adhesive energy of super tattoo gradually decreased (Figure 4b). This is because an increase in fiber density will result in larger encapsulation ability of PSBMA‐DES gel and bigger surface roughness, thus reducing the conformal contact between super tattoo and skin. To guarantee the super tattoo possessing sufficient tensile strength to self‐support, a fiber density of 0.5 mg·cm^−2^ was selected for optimal balance (Figure 4c), and the composition of PSBMA‐DES gel was further adjusted for higher adhesion (Figure 4d). PSBMA contains a large number of amphoteric ion pairs, which can form a gel network through the ionic bonding and cross‐linking between the ion pairs. DES, its free ions are conducive to the opening of more cross‐linked ionic bonds, thus reducing its cross‐linking density. At low concentrations of DES, the cohesion of the PSBMA‐DES gel was prominent, while its adhesion was minimal due to the high cross‐linking density. With the increase of DES concentration, more ionic bonds were opened, and its adhesion gradually increased due to the increased dynamic bonding. Excessive DES led to a low cross‐linking density. The viscous products formed by linear PSBMA and DES will leave residues on skin after super tattoo was removed. Besides, the ionic groups in PSBMA, encapsulated by an excessive amount of DES, gradually exhibited reduced adhesion. Super tattoo at a DES concentration of 40 wt % had the best adhesion, with an adhesive force of 120 kPa and an adhesive energy of 0.92 kJ·m^−2^ (Figure 4e). Such super tattoo can tightly adhere to skin, plastic, glass, and metal (Figure 4f), which is superior to EC tattoo.

PSBMA contains a large number of zwitterion pairs, which makes it a good electrolyte as ion migration channel. DES formed by hydrogen bond acceptor choline chloride (ChCl) and hydrogen bond donor glycerol has the advantages of non‐volatile, non‐toxic properties, and good ion conductivity.^[^ ^26^ ^]^ Thus, PSBMA‐DES gel can ensure super tattoo with stable and high electrochemical performances (Figure S6, Supporting Information). With the increase of DES concentration, the ion conductivity of super tattoo increased (Figure 4g,h). When the DES concentration was 40 wt %, ion conductivity can reach 16 ± 0.5 mS·m^−1^ (Figure 3h). Therefore, in addition to breathability and skin‐like tensile property, super tattoo is adhesive to skin and highly ion conductive, which is very suitable for tattoo electronics.

### 2.5 Super Tattoo‐Based Electrodes for Electrophysiology

**Figure 5:**
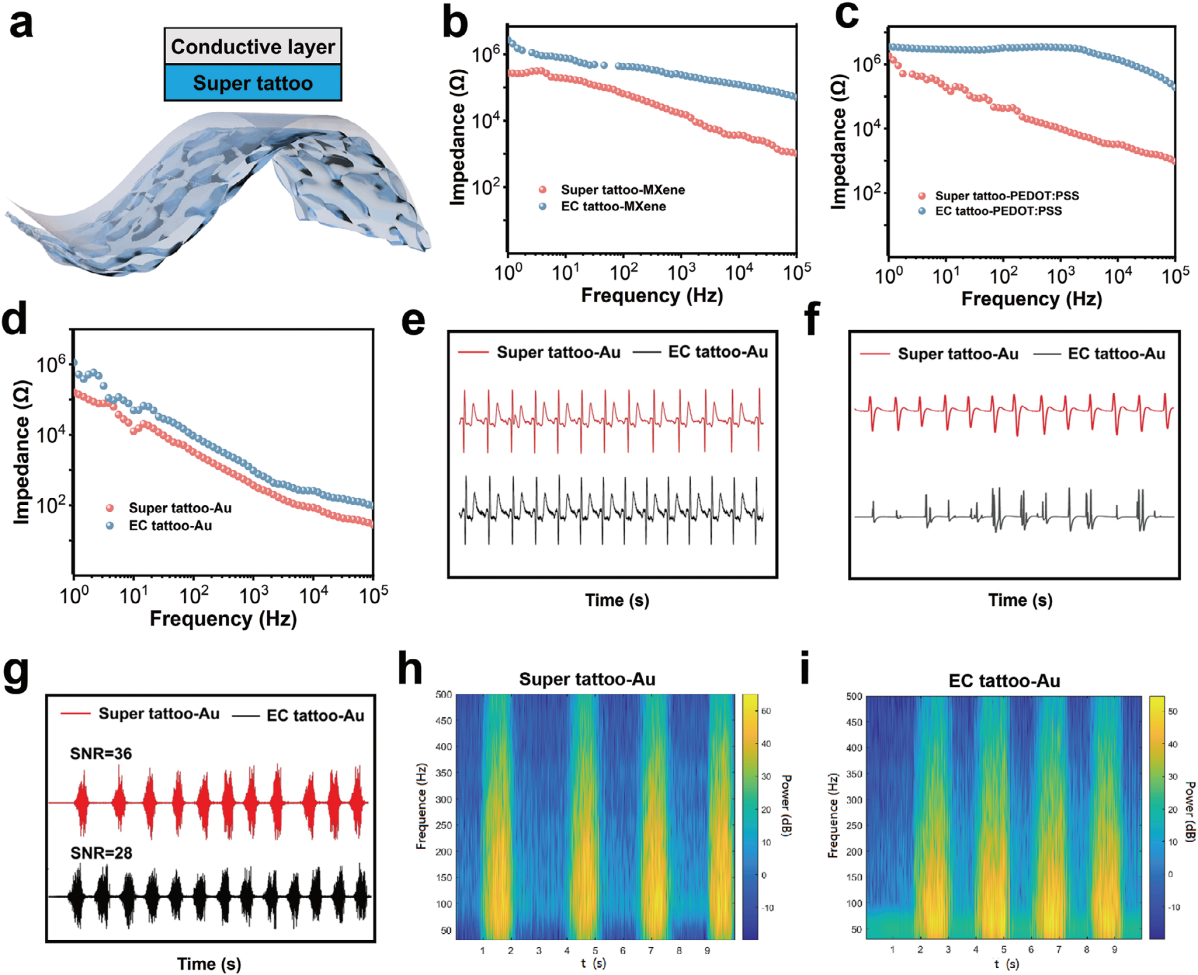
Super tattoo‐based electrodes for electrophysiology. a) Schematic diagram of super tattoo‐based electrodes. (b‐d) Comparison of skin interface impedance of super tattoo‐based electrodes and EC tattoo‐based electrodes, using b) MXene, c) PEDOT:PSS, and d) Au as electrode materials, respectively. e–g) ECG (e), f) EOG, and g) EMG signals collected by super tattoo‐Au electrodes (red) and EC tattoo‐Au electrodes (black). h,i) Comparison of time‐frequency mapsof EMG by h) super tattoo‐Au electrodes and i) EC tattoo‐Au electrodes.

Electrophysiological signals convey abundant information for physical health, emotional analysis and human‐machine interface. Super tattoo, featured in ultrathin, skin‐like mechanical and electrical properties, is an excellent platform for electrophysiological electrodes. MXene, Poly (3,4‐ethylenedioxythiophene)‐poly (styrenesulfonate) (PEDOT:PSS), and Au are three typical electrode materials, which were coated on super tattoo for electrophysiological testing (**Figure** **5**a). First, the electrode was positioned on the skin of upper arm, and the impedance at skin interface was measured using an electrochemical workstation. Super tattoo‐based electrodes exhibited a skin interface impedance that is approximately one order magnitude lower in the range of 10–100 Hz than EC tattoo‐based electrodes, which was coated with same conductive materials (Figure 5b–d). Among above super tattoo‐based electrodes, super tattoo‐Au electrode can reach the lowest skin interface impedance of ≈10^4^ Ω at 10 Hz. These results verified that super tattoo contributes to the features of intimate adhesion on skin, ionic conductivity and certain conformability upon strain. The seamlessly conformal contact with the skin can effectively minimize the skin interface impedance and reduce motion artifacts of signals.

Super tattoo‐Au electrodes were next applied to collect electrophysiological signals from our body. ECG signals measured by super tattoo‐Au electrodes can clearly distinguish representative P, Q, R, S, T peaks, which are comparable to those measured by EC tattoo‐Au electrodes (Figure 5e). However, for the electrophysiological signals that arise accompanied with significant skin deformation, such as electroretinography (EOG) electromyography (EMG), super tattoo‐based electrodes possess more advantages than EC tattoo‐based electrodes in terms of signal noise level. EOG is an electrical signal generated during the eye movement, and reflect eye health status. EOG signals measured by super tattoo‐Au electrodes exhibited clearer triangular peaks with more uniform amplitudes than those by EC tattoo‐Au electrodes (Figure 5f). EMG measures electrical activity of muscles and reflect the muscle health, which is often disturbed by motion‐artifact. Electrodes were used for collecting upper arm EMG signals when the volunteer held the same grid strength (Figure 5g). Super tattoo‐Au electrodes exhibited a higher signal‐to‐noise ratio (SNR) value (SNR = 36) than EC tattoo‐Au electrodes (SNR = 28). From time‐frequency maps (Figure 5h,i), when the arm was relaxed, super tattoo‐Au electrodes exhibited lower motion artifacts than that of EC tattoo‐Au electrodes in the noise range of 50–100 Hz. This indicated that super tattoo‐Au electrodes were less susceptible to noise interference during EMG collection and had better resistance to motion artifacts. This was attributed to the excellent adhesion and skin‐like mechanical properties and toughness of super tattoo substrates, while EC tattoo lacked sufficient adhesion and mechanical strength, which will cause large gaps or even detachment when skin is under large deformation.

### 2.6 Swallowing Training by Super Tattoo‐Based Electrodes

**Figure 6:**
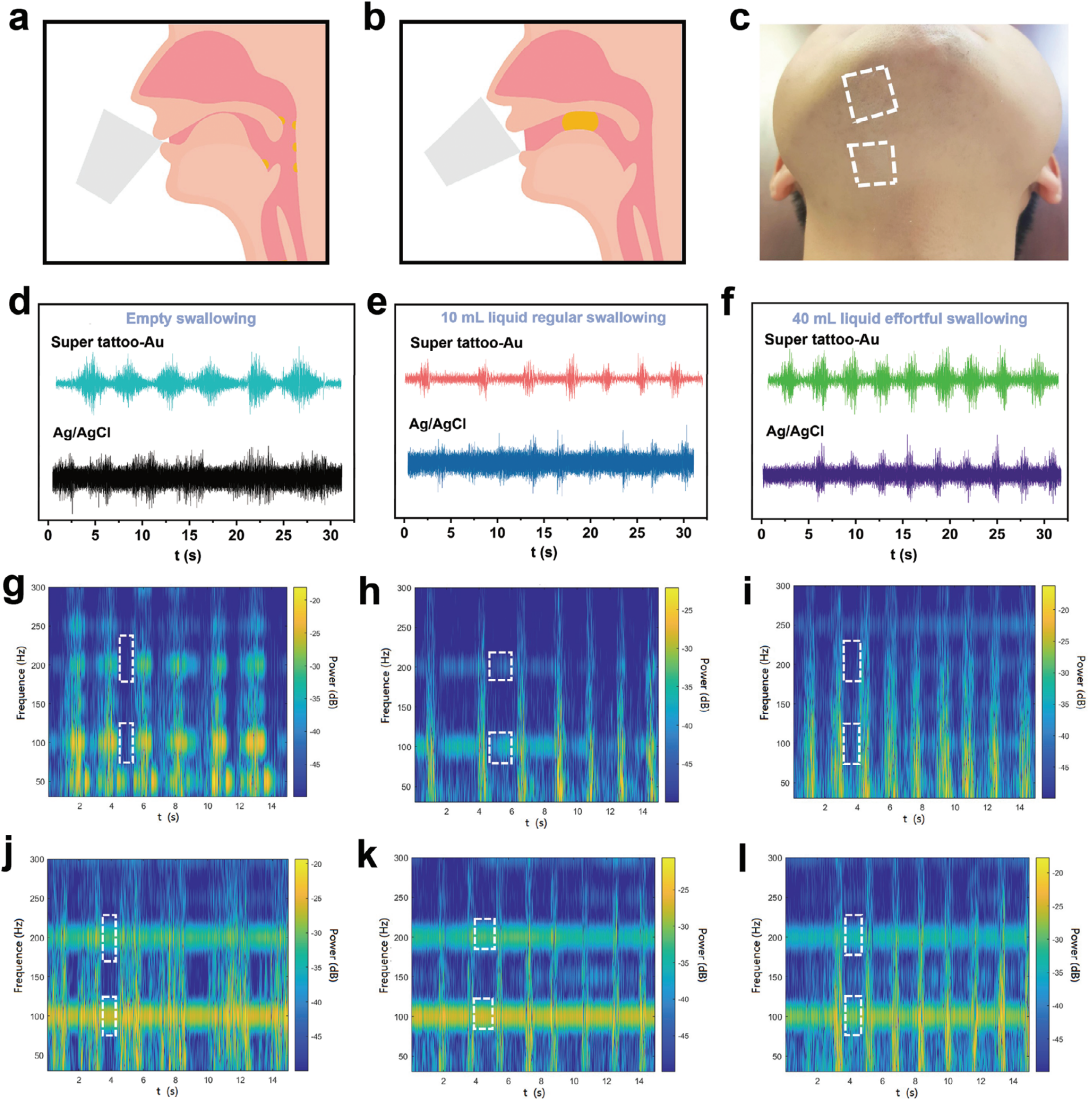
Swallowing training by super tattoo‐based electrodes. a,b) Schematic diagram of muscle movement during swallowing. c) Super tattoo‐Au electrodes adhered to the mandibular triangle. d–f) EMG of empty swallowing (d), e) regular swallowing of 10 mL of liquid, and f) effortful swallowing of 40 mL of liquid collected using super tattoo‐Au electrodes and commercial Ag/AgCl electrodes, respectively. (g‐l) Time‐frequency maps of EMG conversion for g) empty swallowing, h) regular swallowing, and i) effortful swallowing by super tattoo‐Au electrode and for j) empty swallowing, k) regular swallowing, and l) effortful swallowing by Ag/AgCl electrode.

Swallowing is a common human activity in daily life, but successful swallowing requires the precise coordination of over forty pairs of muscles in the head and neck, six pairs of brain nerves, complex circuits in the brainstem, and multiple brain regions.^[^ ^27^ ^]^ Any interruption of these pathways may lead to swallowing disorders. Swallowing disorders are usually caused by various neurological, respiratory, digestive system or diseases, which can interfere with the patient's daily life. If not treated properly, it may lead to serious consequences, such as malnutrition, dehydration, or respiratory system damage or even death due to food inhalation into the lungs. The hyoid muscle located at the head and neck is an important muscle in the swallowing mechanism. As shown in **Figure** **6**a,b, when swallowing reached the pharyngeal stage, the swallowing reflex was triggered, prompting the hyoid muscle to lift the hyoid bone and throat. Meanwhile, the epiglottis closed the trachea to protect the respiratory system, while the food ball was pushed into the esophagus by the concerted action of the hyoid muscle group, commencing from the pharynx. Monitoring the function of the hyoid muscle offers insights into the patient's pathological changes. At present, rehabilitation training for dysphagia mainly relies on expensive biofeedback equipment, such as puncture electrode, oral manometer or endoscope to detect head/neck movement, which will cause certain injuries and discomfort to patients. Also, patients need to go to hospital frequently for treatment, which is usually expensive and inconvenient.

Skin electrodes have the advantages of non‐invasiveness and ease of use. Nevertheless, commercial electrodes, such as Ag/AgCl, encounter significant challenges when used in swallowing activities, due to the considerable skin deformation caused by neck curvature and contraction or stretching of the pharyngeal muscles. Commercial electrodes have poor skin conformability or even detachment, bringing huge interferences to the accurate acquisition of swallowing EMG. Moreover, thicker thickness and lack of breathability of commercial electrodes cause severe discomfort for the tester.^[^ ^28^ ^]^ In contrast, our super tattoo‐based electrodes exhibited breathability, adhesiveness, and conformability to skin, even under conditions of skin deformation. This made them potentially ideal for acquiring swallowing EMG with high fidelity. Super tattoo‐Au electrodes were adhered to the junction of the mandibular triangle and neck (Figure 6c). During the testing, the volunteer swallows 10 and 40 mL of water respectively, along with performing empty swallowing, a commonly employed exercise in clinical swallowing disorder recovery training. EMG signals collected by super tattoo‐Au electrodes exhibited lower motion artifacts than those by commercial electrodes at all these swallowing tests, clearly showing the characteristic peaks (Figure 6d–f). Empty swallowing of 40 mL of water was associated with a longer swallowing duration and a more prominent amplitude compared to regular swallowing of 10 mL of water, while empty swallowing exhibited the longest swallowing time and presents a longer spindle shape (Figure 6g–l). This may be due to the absence of food stimulating the swallowing reflex during empty swallowing, resulting in delayed response of the hyoid muscle. These results indicated that EMG signals collected by super tattoo‐Au electrodes can provide clinically useful information in identifying swallowing disorders and provide effective feedback for the development of effective swallowing rehabilitation plans.

## 3 Conclusion

In summary, we mimic human skin and synthesize an ultra‐thin skin‐like super tattoo substrate (≈2 µm) that consists of electrospun silk fibroin fibers as support and polysulfobetaine (PSBMA) – deep eutectic solvent (DES) gel as matrix. Such structure design together with plasticizing effect by water absorption of PSBMA and DES on silk fibroin, endows skin‐like tear resistance and strain stiffening characteristics to the super tattoo, with tear energy (5.4 kJ·m^−2^) and toughness (1.3 ± 0.2 MJ·m^−3^). In addition, the synergistic interaction between electronspun silk fibroin fibers and PSBMA‐DES gel matrix enables super tattoo with adhesion (123 kPa), air permeability and ion conductivity. Super tattoo was then applied as a general platform, fabricating for breathable and conformable tattoo electrodes for electrophysiology. Compared with EC tattoo‐based electrodes, super tattoo‐based electrodes have the advantages of lower skin interface impedance (≈10^4^ Ω), higher SNR, and lower motion artifacts. Therefore, super tattoo‐based electrodes are capable of accurately and imperceivably acquiring swallowing EMG signals, potentially serving as a valuable tool for guiding rehabilitation training for patients with swallowing disorders.

## 4 Experimental Section

### Materials

The silkworm cocoons without pupae was purchased from Northwest Silkworm Base, the sodium bicarbonate (99.99%), choline chloride (ChCl, 99%) and the [2‐(methacryloyloxy) ethyl] dimethyl‐(3‐sulfopropyl) ammonium hydroxide (SBMA, ≥97%) were obtained from Aladdin Industrial Corporation, 2‐hydroxy‐2‐methyl‐1‐ [4‐ (2‐hydroxyethoxy) phenyl] −1‐acetone (Irgacure 2959, 98%) was obtained from Thermo Fisher Scientific, the formic acid (99%), glycerin (≥99.5%) and the N, N′‐ Methylenebisacrylamide (MBA, 99%) were obtained from Innochem. PEDOT:PSS was purchased from Han Feng.

### Fabrication of Super Tattoo and Super Tattoo‐Based Electrodes

Freeze‐dry the silk fibroin solution to obtain regenerated silk fibroin powder and dissolve it formic acid to prepare the silk fibroin precursor solution. Add glycerol and stir for 1 h to improve the stability of silk fibroin. Electrospin silk fibroin fibers with a voltage of 10 kV in an ambient environment at humidity of 30% (RH). A cut copper foil frame was used as the receiving substrate. By mixing the hydrogen bond acceptor ChCl with the hydrogen bond donor glycerol in a molar ratio of 1:2 and stirring at 60 °C for 2 h, a transparent and clear liquid deep eutectic solvent (DES) was obtained. 6 g SBMA, 2 mg MBA, 5 g DES, 0.01 g 2959 photoinitiator (Irgacure 2959) and 18 mL water were stirred and mixed evenly to obtain the gel precursor solution. The electrospun silk fibroin fiber film was then immersed in gel precursor solution, and photopolymerized in glove box with 365 nm ultraviolet light for 12 min to form PSBMA DES gel matrix. Super tattoo was thus fabricated with electrospun silk fibroin fibers as scaffold and PSBMA DES gel as matrix.

PEDOT:PSS solution was prepared simply by mixing 1% PEDOT:PSS aqueous solution (Clevios PH 1000) and 0.5–1% glycerol. Super tattoo was first soaked in above solution for 1 min, and then the coated super tattoo was dried under ambient conditions (25 °C and 20% humidity).

MXene suspension was prepared according to chemical etching method, and super tattoo was immersed in 2.5 mg mL^−1^ MXene suspension to obtain super tattoo‐MXene electrode, followed by drying under ambient conditions for 15 min.

The super tattoo‐Au electrode was obtained by evaporating 70 nm Au thin film onto super tattoo via thermal evaporation.

By coating with MXene, PEDOT:PSS solution and evaporated Au onto super tattoo as a universal substrate, electronic tattoo can be achieved and applied in bioelectronics such as for electrophysiological signal acquisition.

### Characterization of Super Tattoo

The electrochemical impedance spectroscopy (EIS) measurements were conducted using a CHI600e electrochemical workstation (CH Instruments). The transparency of the thin film was tested by an ultraviolet‐visible spectrometer (UV‐2450). The FTIR spectrum were collected by the IRAffinity‐1 Fourier Transform Infrared Spectrometer (IRAffinity‐1) spectrometer.

### Mechanical Tests on Super Tattoo

The tensile mechanical properties were directly characterized on an ultrathin super tattoo which was with a length of 10 mm and a width of 8 mm and fixed on a 20 mm × 20 mm copper frame window onto a Mark‐10 testing machine. Then the sample was clamped on a stretching bracket. The contact area between the suspended film and the target adhesive substrate is 80 mm^2^. The Mark‐10 testing machine moves at a separation speed of 20 mm ·min^−1^ until the sample separates from the adhesive substrate. For single‐edge notch tension (SENT) tests, a notch was cut by a blade on one side of the sample, and tensile testing was performed at a tensile speed of 20 mm ·min^− 1^. The fracture energy (Γ) was calculated by the following equation:

(1) $$\Gamma =\frac{6Wc}{\surd \lambda_{c}}$$

where λ_c_ is the fracture strain rate of notched samples, c is the length of the notch, and W is the stress‐strain curve integral of notched specimen with the same size stretching to the same strain of notched specimen.^[^ ^12^, ^29^ ^]^

### Water Vapor Transmission Test

The water vapor transmission rate (WVTR) was tested at 25 °C and 45% humidity on the basis of standard ASTM E96. The tests were performed by periodically measuring the weight loss of water in a bottle where the bottle mouth was covered by different thin films.^[^ ^30^ ^]^

(2) $$WVTR\;=\;\frac{\Delta m}{A*T}$$

where ∆m is the weight change of the water bottle, T is the time during the weight change period, and A is the area of bottle mouth.

### Electrophysiological Measurements

Two working electrodes were placed on forearm, and the reference electrode was placed on the back of wrist for arm EMG. Two working electrodes were placed on submental area, and the reference electrode was placed on the postauricular mastoid process for swallowing EMG. The electrodes were connected with commercial signal recording equipment BYB SpikerBox (Backyard Brain). For ECG, two working electrodes and reference electrodes were placed on arm by using the Einthoven triangle principle. During EOG measurement, the working electrodes were placing on the forehead and lower eyelids, respectively.

It was confirmed that the human experiments in this study were approved by the Human Research Ethics Review Committee for Beijing Normal University, and all participants were informed and signed informed consent forms. The ethics number of the experiment is IRB_B_0026 2 021 001.

## Conflict of Interest

The authors declare no conflict of interest.

## Author Contributions

C.L. and Z.T. contributed equally to this work. N.L. and C.L. conceived the original idea and designed the experiments. C.L. performed the experiments. Z.T., X.S., Y.Z., Y.Z., Z.Z., W.Z., J.Q., Y.W., and X.W. participated in the partial measurements. C.L. and Z.T. wrote the manuscript. Z.T. drew the schematic diagrams. N.L. analyzed the data. N.L. and Z.T. supervised the study. N.L. and Z.T. revised the manuscript. All the authors took part in the discussion and writing.

## Supporting information

Supporting Information

## Data Availability

The data that support the findings of this study are available from the corresponding author upon reasonable request.
