# Supplementary material for: Breathable, Adhesive, and Biomimetic Skin‐Like Super Tattoo
Source: Adv Sci (Weinh). 2024 Aug 29;11(40):2406706. doi: 10.1002/advs.202406706 (PMC11515898; doi:10.1002/advs.202406706)
Supplement: Supplementary file 1 — Supporting Information [file ADVS-11-2406706-s001.docx]

Supporting information

Breathable, Adhesive and Biomimetic Skin-Like Super Tattoo

Chuqi Li^1,2^^†^, Zhiyuan Tan^1†^, Xiaohu Shi^1^, Dekui Song^1^, Yan Zhao^1^, Yan Zhang^1^, Zihan Zhao^1^, Weifeng Zhang^1^, Jiongyang Qi^1^, Yifang Wang^1^, Xin Wang^1^, Zhenquan Tan^2*^and Nan Liu^1*^

^1^Beijing Key Laboratory of Energy Conversion and Storage Materials, College of Chemistry, Beijing Normal University, Beijing 100875, P. R. China

^2^State Key Laboratory of Fine Chemicals, Panjin Branch of School of Chemical Engineering, Dalian University of Technology, Panjin 124221 Liaoning, China

* Corresponding author: E-mail: nanliu@bnu.edu.cn; tanzq@dlut.edu.cn

^†^C. L. and Z. T. contributed equally to this work.

Fabrication of regenerated silk fibroin protein

Regenerated silk fibroin are prepared from degummed Bombyx mori silk fibers as reported.

Degumming:

1. Cut and shred the silk cocoon, and then soak it in a 0.5 wt % NaHCO_3_ aqueous solution.
2. Heat and boil it for 30 minutes.
3. Remove it, and rinse it repeatedly in deionized water three times.
4. Repeat above steps twice.
5. Place the washed silk fibroin in a 60 ℃ drying oven overnight to dry the water, and obtain the original silk fibroin that have removed the sericin.

Dissolution:

1. 5g degummed original silk fibroin are dissolved in 25 mL LiBr solution (9.3 mol L^-1^ , related to LiBr) for preparation.
2. Heat in a 60 ℃ water bath for 1 hour, and continuously stir it meantime.
3. Cool to room temperature, and obtain yellow viscous liquid.

Dialysis:

1. Put the dissolved silk fibroin solution into a dialysis bag.
2. Soak it in deionized water, and store it in refrigerator at 4 ℃.
3. Change the water every 4 hours, and dialyze for 4-5 days to remove ions from the solution.
4. Place the dialyzed silk fibroin solution in a centrifuge at 7500 rpm for 8 minutes to precipitate impurities.
5. Repeat twice to obtain the supernatant and obtain silk fibroin solution (5 wt %, related to silk fibroin).
6. Freeze-dry the solution using a vacuum freeze dryer at -60 ℃ and 30 Pa to obtain regenerated silk fibroin powder for storage.


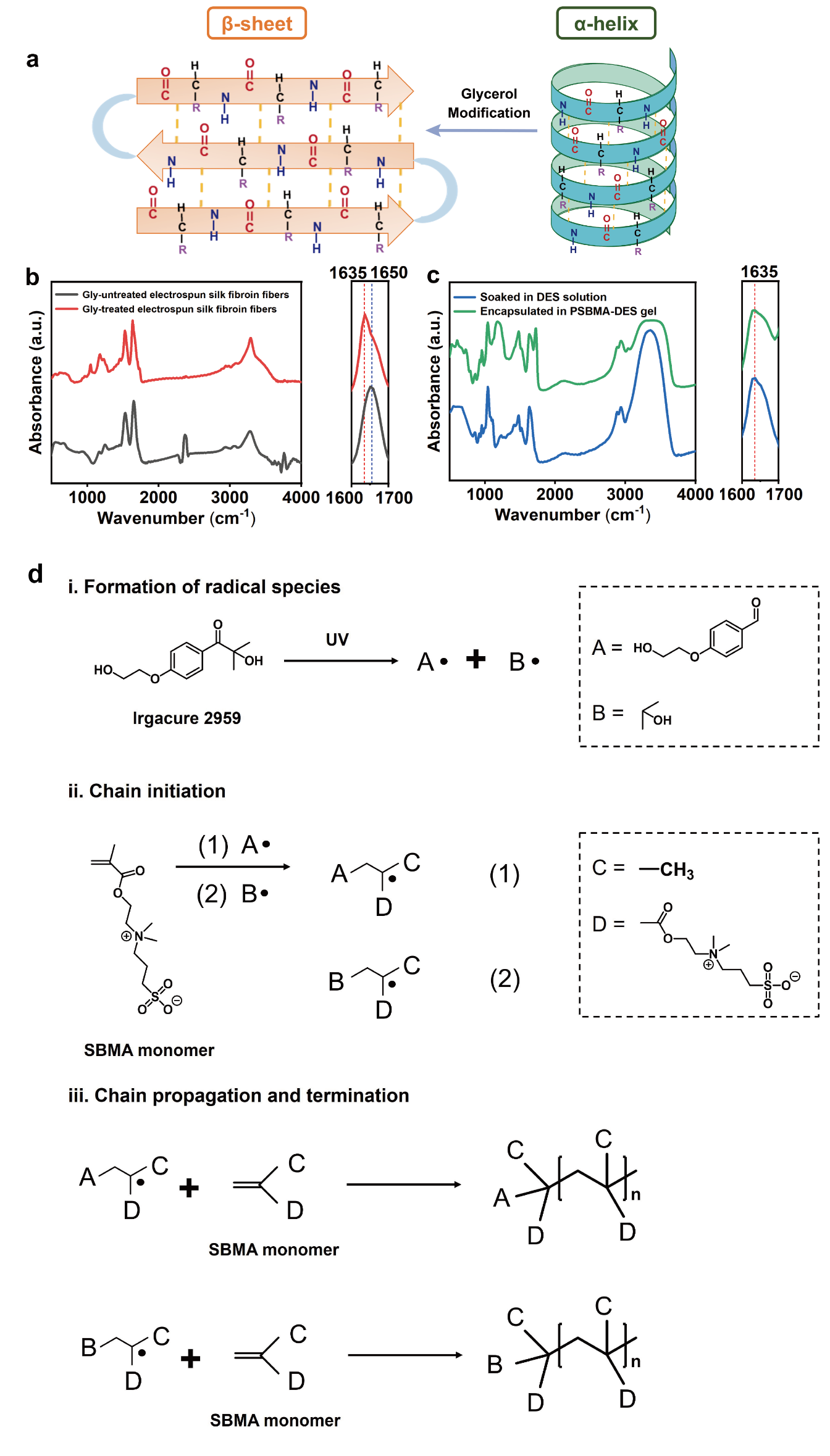


**Figure S1** (a) Schematic diagram of the mechanism of glycerol treatment to improve the stability of silk fibroin. (b) The FTIR spectra of Gly-untreated electrospun silk fibroin fibers (red) and Gly-treated electrospun silk fibroin fibers (black). (c) The FTIR spectra of Gly-treated electrospun silk fibroin fibers soaked in DES solution (blue) and Gly-treated electrospun silk fibroin fibers encapsulated in PSBMA-DES gel matrix (green). (d) The synthesis mechanism of SBMA monomers polymerization induced by Irgacure 2959.

Durability and mechanical properties of silk fibroin are largely determined by its secondary structure, where β-sheet structure formed by hydrogen bonding between the carboxyl group (-C=O) and amino group (-NH) of amino acids on the molecular chain, rotating and folding at 180°, has better air stability and mechanical strength compared to the loose amorphous structures. Electrospun silk fibroin fibers that electrospinned from different electrospinning solution were ground into powder for Fourier transform infrared spectrum (FTIR) test. As shown in the Figure S1b, compared to the FTIR spectrum of electrospun silk fibroin fiber, whose electrospinning solution was not treated with glycerol (Gly-untreated electrospun silk fibroin fibers), the amide I characteristic absorption band of the one whose electrospinning solution was treated with glycerol (Gly-treated electrospun silk fibroin fibers), shifted from 1650 cm^-1^ to 1635 cm^-1^ (Figure S1b), indicating that more amorphous structures in the secondary structure were transformed into β-sheet through glycerol treatment (Figure S1a). Additionally, the Gly-treated electrospun silk fibroin fibers whether soaked in DES solution or encapsulated in PSBMA-DES gel matrix, the peak positions of amide I characteristic absorption bands remained basically unchanged (Figure S1c), indicating that Gly-treated electrospun silk fibroin fibers are able to keep a high and greatly stable β-sheet content.

After electrospinning, the electrospun silk fibroin fiber film need to be immersed in gel precursor solution, and then photopolymerized in glove box with 365 nm ultraviolet light for 12 min to form PSBMA-DES gel. In this photopolymerization process, Irgacure 2959 is exposed to specific wavelengths of UV light to dissociate and generate free radicals. The double bond in the SBMA structure is induced to open for polymerization reaction, forming polymer chains (Figure S1d). The generated polymer chain forms polymer network through the chemical cross-linking sites provided by MBA, and finally forms gel matrix.


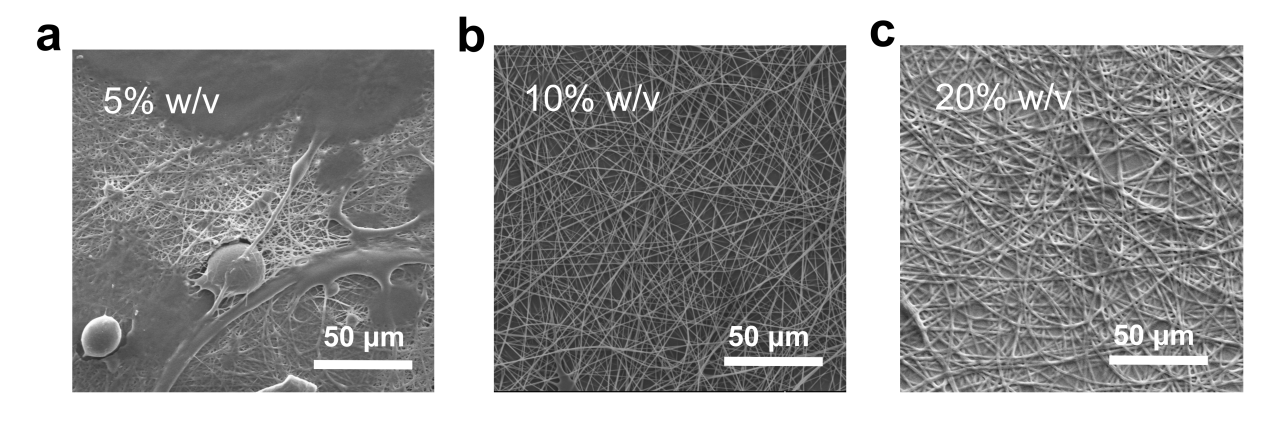


**Figure S2.** Scanning electron microscopy (SEM) images of the electrospun silk fibroin fibers film electrospinned by electrospinning solution whose concentration (related to silk fibroin) is (a) 5 % w/v, (b) 10 % w/v, (c) 20 % w/v.

The morphology of the silk fibroin fibers film electrospinned by fibroin formic acid electrospinning solution with different concentrations (the following concentrations represent the content of silk fibroin in the electrospinning solution) was observed using scanning electron microscope (SEM). As shown in Figure S2a-b, when the concentration is 5 % w/v, the obtained film fibers have poor uniformity and contain a large number of silk fibroin beads. This is due to the low concentration of silk fibroin and the low viscosity of the electrospinning solution. When the solution jet is stretched by an electric field, the molecular chains are not entangled enough to effectively resist external forces and break. At the same time, due to the viscoelastic effect of silk fibroin molecular chains, they tend to contract, leading to the aggregation of molecular chains and ultimately forming silk fibroin beads. When the concentration increases to 10 % w/v, the viscosity of the electrospinning solution increases to a certain extent, and the molecular chains are appropriately entangled, and the tensile force is relatively uniform. The jet undergoes whipping in the electric field due to surface charges and electric field forces. After the solvent evaporates, it solidifies into fibers with relatively uniform diameter. However, when the concentration increases to 20 % w/v, the viscosity of the electrospinning solution becomes higher, leading to the molecular chains of the electrospinning solution are highly entangled, and the obtained silk fibroin fiber diameter formed under the electric field force is too large. And it results the electrospun silk fibroin fibers film is too thick and dense, which leads to poor breathability of the film. Considering the uniformity and breathability of the film, it is better to use 10 % w/v electrospinning solution for electrospinning.


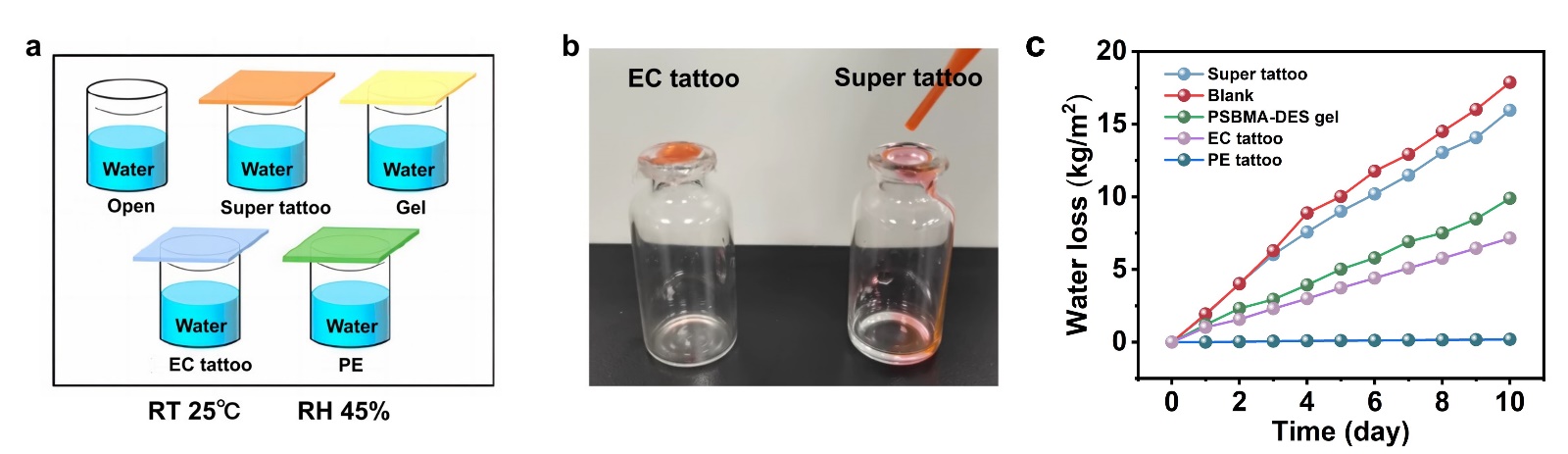


**Figure S3.** (a) Schematic diagram of water vapor transmission experiment. (b) Comparison of water permeability between commercial EC tattoo and Super tattoo. (c) Mass changes of water vapor transmitted by different thin films over time.

Place commercial EC tattoo and super tattoo to the mouth of bottle respectively, and then slowly add water droplets slowly while observing the penetration of different tattoo at 25 ℃ and 45 % humidity. Figure S3b shows that water droplets can easily pass through the super tattoo and flow into the bottom of the bottle, while EC tattoo hinder the penetration of water droplets, causing water to accumulate at the bottle mouth.


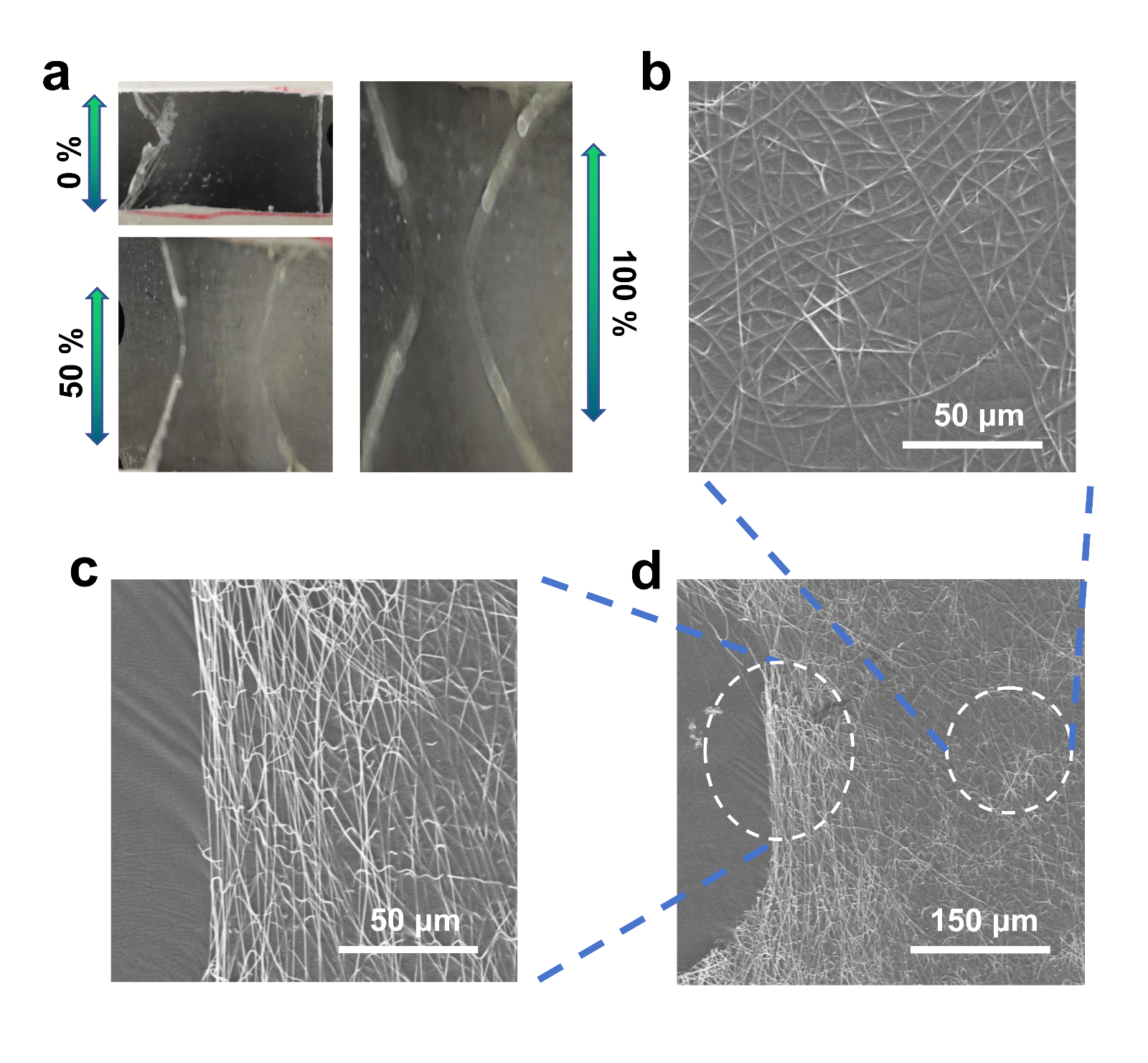


**Figure S4** (a) Tensile photos of a notched electrospun silk fibroin fiber film. (b) SEM images of the interior, (c) edge, and (d) overall of the notched electrospun silk fibroin fibers film during stretching.


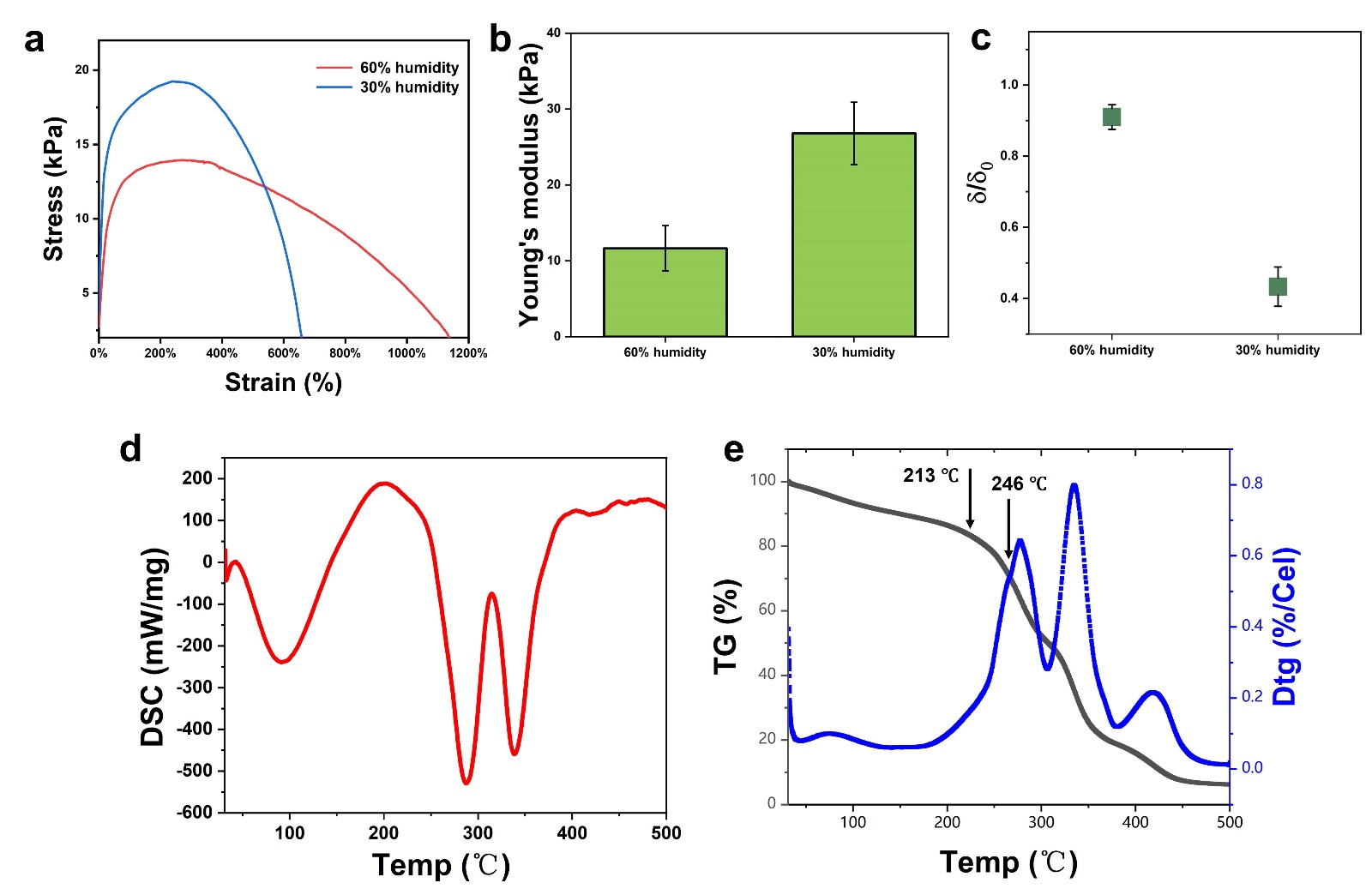


**Figure S5** (a) Stress-strain curve of PSBMA-DES gel under different humidity conditions. (b) Corresponding Young’s modulus of PSBMA-DES gel. (c) Ionic conductivity under different humidity conditions. (d) DSC, (e) TG -DTG patterns of PSBMA-DES gel.

According to the Figure S5d and S5e, the weight loss process of gel can be divided into three stages. The first weight-loss stage is about 31~213 ℃, and the weight-loss platform is wide and gradual. In this stage, free water and intermediate water in gel are lost, which is a continuous process. A significant decrease in mass occurred at 246 ℃, indicating a significant structural change in PSBMA. Compared to the first stage, the weight loss occurred faster in this stage.


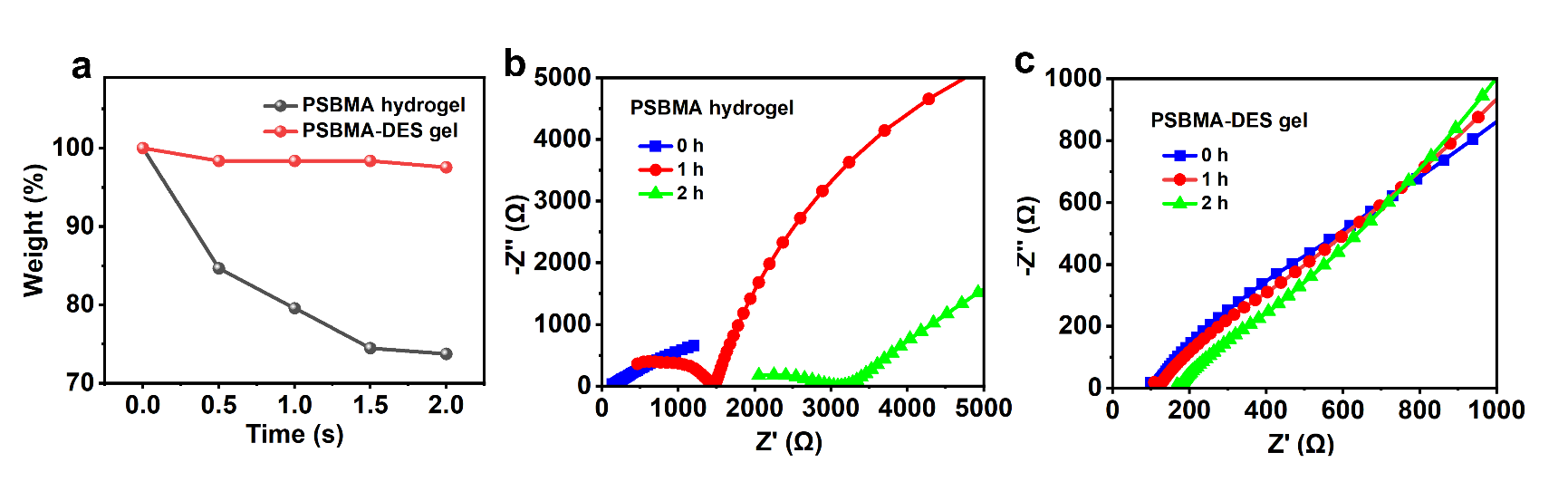


**Figure S6** (a) The weight change of PSBMA-DES gel and PSBMA hydrogel in dry environment. (b) The Nyquist plots of PSBMA hydrogel placed in a dry environment for different durations. (c) The Nyquist plots of PSBMA-DES gel placed in a dry environment for different durations.

As shown in Figure S6a, put PSBMA-DES gel and pure PSBMA hydrogel in a drying oven with a humidity of 10 % and at a temperature of 36 ℃ to record the weight change of gel. PSBMA hydrogel quickly lost 34 % of its moisture within 2h. Due to the loss of water, and the ion conductivity of PSBMA hydrogel greatly decreases. As shown in the Figure S6b, its ion conductivity became 100 times lower than the ion conductivity of the original PSBMA hydrogel after 2h. However, the weight of PSBMA-DES gel remained basically stable throughout the whole test process, that the weight change of PSBMA-DES gel was only 1 %, and its ion conductivity only slightly shifted (Figure S6c).


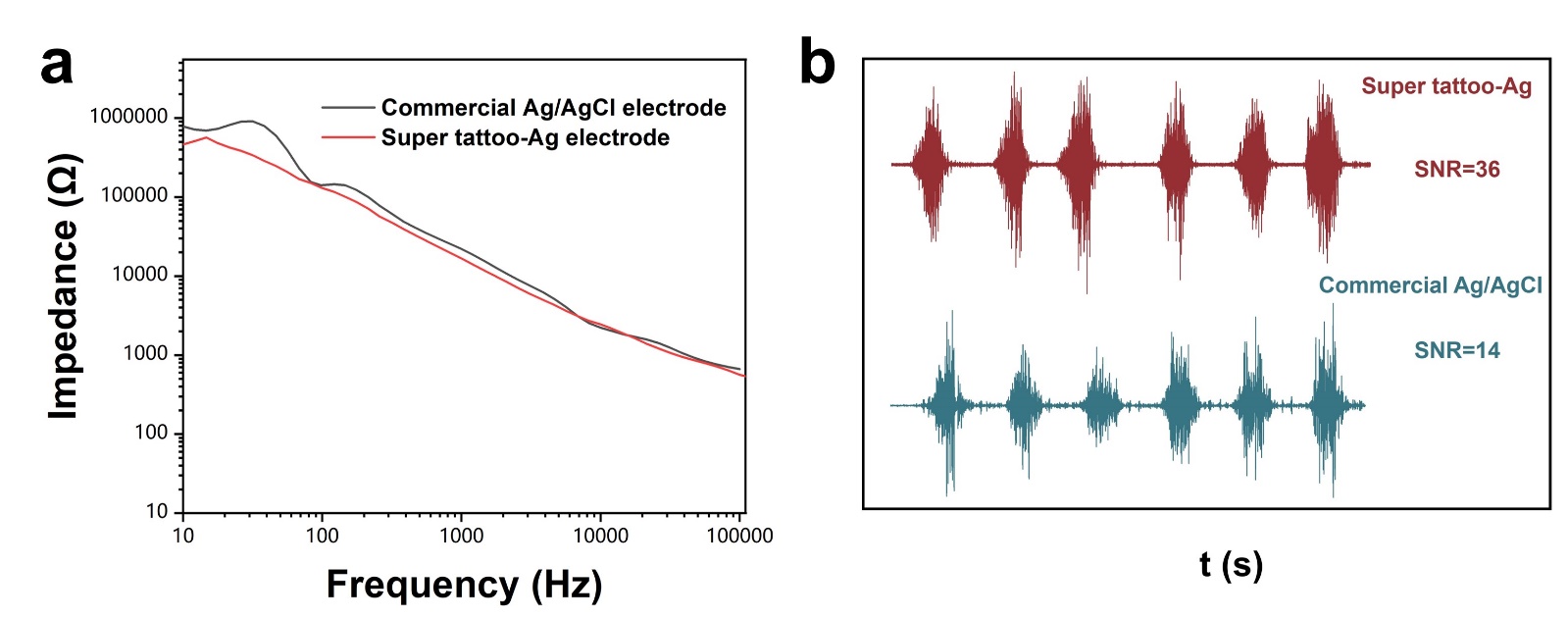


**Figure S7** (a) Skin interfacial impedance of the super tattoo-Ag electrodes in comparison with commercial Ag/AgCl electrode. (b) EMG signals recorded by super tattoo-Ag electrode and commercial Ag/AgCl electrode and corresponding SNR values.
